# A Scoping Review of Clinical Utility from the Montreal Cognitive Assessment Memory Index Score

**DOI:** 10.1177/08919887251366698

**Published:** 2025-08-18

**Authors:** Oscar R. Kronenberger, Alyssa N. Kaser, Jeff Schaffert, Vishal J. Thakkar, William Goette, Christian LoBue, Laura H. Lacritz

**Affiliations:** 1 Department of Psychiatry, 12334 University of Texas Southwestern Medical Center, Dallas, TX, USA; 2 Department of Neurological Surgery, 12334 University of Texas Southwestern Medical Center, Dallas, TX, USA; 3 Department of Neurology, 12334 University of Texas Southwestern Medical Center, Dallas, TX, USA

**Keywords:** montreal cognitive assessment, memory index score, neurodegenerative dementia, cognitive decline, mild cognitive impairment, Alzheimer’s disease

## Abstract

**Objective:**

The Montreal Cognitive Assessment (MoCA) Memory Index Score (MIS) is a supplemental assessment of memory composed of word list delayed free-recall followed by step-down category cued- and multiple-choice cued-recall. This paper reviews the MIS literature within Alzheimer’s and other neurodegenerative dementias to synthesize evidence regarding its clinical utility, identify gaps, and inform future research directions.

**Method:**

We searched electronic databases of OVID Medline, Embase, PsycINFO, and PubMed from 2014, when the MIS was first described, to July 2025. Peer-reviewed studies that reported data on the diagnostic or prognostic utility of the MIS in assessing neurodegenerative dementia populations were included.

**Results:**

We screened 278 articles, and 14 were included in the review. The current literature includes limited reporting on the diagnostic or prognostic utility of the MIS and is characterized by minimal diversity of samples and non-rigorous validation methods. Initial findings are promising and suggestive of incremental validity over the MoCA total score for identifying episodic memory impairment and therefore aiding in differentiation of suspected dementia etiology. However, evidence is insubstantial for the MIS as a tool for predicting progression and additional research is needed to evaluate the incremental validity of the MIS over the conventional MoCA five-word recall score.

**Conclusions:**

Large literature gaps exist regarding the clinical utility of the MIS within neurodegenerative dementias. Additional research exploring the psychometric properties of the MIS using diverse samples with rigorous validation methods is needed to better inform its application.

The Montreal Cognitive Assessment (MoCA) is a cognitive screening instrument developed to detect mild cognitive impairment (MCI), which was supported by initial findings demonstrating that the MoCA total score (TS) had excellent sensitivity (90%) and good specificity (87%) in the detection of MCI.^1^ The MoCA-TS ranges from 0 to 30 points and assesses various cognitive domains, including visuospatial and executive function (5 points), naming (3 points), attention (6 points), language (3 points), abstraction (2 points), orientation (6 points), and delayed free-recall (5 points). The MoCA has steadily grown in popularity as a cognitive screener across clinical and research settings due to its cost-free availability, time-efficient administration, and effectiveness in detecting MCI, outperforming other cognitive screening instruments such as the Mini-Mental State Examination (MMSE).^2,3^ Julayanont and Nasreddine (2017) claimed the MoCA’s effectiveness in detecting amnestic MCI (aMCI) may be attributable to the MoCA’s delayed-recall component, which uses more words (5 vs 3), fewer opportunities for learning (2 vs up to 6), and a longer delay (5 minutes vs 2 minutes) than the MMSE.^4^

The Memory Index Score (MIS) was first described by Julayanont and colleagues (2014) along with 5 additional MoCA index scores (Table 1). The MIS was the only index added to the record form in later versions as a supplemental approach to quantify correct recall of items under cued and multiple choice prompting that does not contribute to the TS.^5^ After 2 learning trials and an approximate five-minute delay, individuals are asked to recall as many of the words as they can, and for any words that they are unable to recall freely, they are provided with step-down cues intended to facilitate retrieval of the remaining word(s). For each of the 5 words, 3 points are awarded for each word freely recalled, 2 points for words recalled after being given a category cue, one point for a correct response to a multiple-choice cue, and zero points if no successful recall is made. Consequently, scores for the MIS can theoretically range from 0 (ie, no words recalled after any cues) to 15 (ie, all 5 words freely recalled without any need for cues), but the forced-choice nature of the cued items results in a chance performance of 2 or 3 out of 15. The developers of the MIS suggested that it may aid in distinguishing memory impairment due to a primary encoding deficit (ie, no improvement with cueing) vs a retrieval memory deficit (ie, improvement with cueing), adding incremental validity to the MoCA.^4,5^

**Table 1.** Domain-specific MoCA Indices as Described by Julayanont et al (2014).^5^

Domain	Range	MoCA items included (maximum points per item)
Memory	0 - 15	Free recall (3 points/word), category cue (2 points/word), multiple-choice cue (1 point/word)
Executive	0 - 13	Trail-making (1), clock drawing (3), digit spans (2), letter-A tapping (1), serial-7 subtractions (3), letter fluency (1), abstraction (2)
Visuospatial	0 - 7	Cube copy (1), clock drawing (3), naming (3)
Language	0 - 6	Naming (3), sentence repetition (2), letter fluency (1)
Attention	0 - 18	Digit spans (2), letter-A tapping (1), serial-7 subtractions (3), sentence repetition (2), immediate recall (10)
Orientation	0 - 6	Date (1), month (1), year (1), day (1), place (1), city/island (1)

*Note*: MoCA = Montreal Cognitive Assessment.

Julayanont and colleagues (2014) suggested that the addition of cued memory performance within the MIS may help assess underlying neurodegenerative progression.^5^ They posited that in the early stages of Alzheimer’s disease (AD), executive functions compensate for hippocampal dysfunction, resulting in benefit from cues to aid with memory recall. As the disease progresses, executive regions are increasingly affected, and memory encoding as well as retrieval performance decline. Therefore, encoding deficits are indicative of more advanced disease progression and an increased likelihood of conversion from MCI to AD. Supporting this theory, research suggests that encoding deficits are the primary characteristic of aMCI and thus AD risk.^6^ Further, impaired cued-recall performance is more closely associated with neuropathological AD changes and more accurately classifies aMCI to AD converters than free-recall performance.^7,8^ Therefore, the incorporation of cued-recall by the MIS may provide valuable prognostic information with those at the highest risk of AD conversion performing poorly.

In addition to this prognostic use, the MIS may have additional clinical value such as classifying individuals into stages of cognitive decline and informing inference about probable dementia etiology. Delayed word list recall tasks, such as the California Verbal Learning Test, Rey Auditory Verbal Learning Test, and Hopkins Verbal Learning Test, are instrumental to the neuropsychological assessment of neurodegenerative dementias, and tasks that assess the multidimensional nature of memory deficits (ie, encoding, consolidation, and retrieval) are particularly useful in the assessment of amnestic disorders.^3,9-12^ In AD, word list delayed free and cued-recall tasks have shown strong diagnostic accuracy, such as differentiating those with AD from healthy controls, suggesting that it may serve as a potential cognitive marker of AD.^13^ Further, performance on free and cued-recall tasks can aid in differentiating between various forms of neurodegenerative disease.^14,15^ For example, Van Liew et al. (2016) found that individuals with Huntington’s disease (HD) and those with AD had poorer free recall than normal controls but those with HD showed greater improvement with cues (characteristic of a retrieval deficit) than those with AD (characteristic of an encoding deficit).^16^ Given the well-established use of the MoCA and the theoretical advantages of the MIS, the potential incremental clinical utility of the MIS warrants further evaluation as it could improve the efficacy of MoCA assessment without the need for additional time or resources.

While the step-down retrieval cues of the MIS are not required to derive the MoCA TS, the MIS has been added to the standard MoCA 8.x record form as a supplemental measure, leaving researchers and clinicians with the decision to administer the cued-items and interpret this score.^5^ However, its psychometric properties and clinical utility remain unclear. Clinicians and researchers would benefit from knowing the scope of existing evidence evaluating the MIS. Yet, to our knowledge, no review exists. Therefore, the current paper aims to survey the empirical literature on the utility of the MIS within neurodegenerative dementia populations. The research reviewed is summarized with respect to the following 4 broad questions that clinicians and researchers are likely to have: (1) whether the available evidence suggests that the MIS is useful above and beyond the MoCA-TS and MoCA free-recall score (ie, has incremental validity), (2) what applications of the MIS are empirically supported (ie, predicting dementia progression, differentiating dementia syndromes, etc.), (3) what methodological themes, limitations, and gaps exist, and (4) what future research is needed to augment understanding of the clinical utility of this supplemental score.

## Methods

The Preferred Reporting Items for Systematic Reviews and Meta-Analyses (PRISMA) scoping review guidelines were followed.^17^ Review protocols were not pre-registered. A medical librarian was consulted to ensure a comprehensive database search strategy. Following pilot testing in the databases, an a priori search protocol was developed.

### Search Strategy

OVID Medline, Embase, PsycINFO and PubMed were searched for publications between January 2014 to July of 2025. Results were limited to articles in the English language published since 2014, as the MIS was first described at this time.^5^ Boolean operators (AND, OR, NOT) with a combination of Medical Subject Headings (MeSH) and search terms were used (see Supplemental Table 1 for the full list of search criteria). A manual search of reference sections of discovered articles was also performed to identify additional articles possibly omitted during the database inquiry process.

### Selection Criteria and Screening

Articles were included if they were: (1) peer-reviewed, (2) utilized the MIS as defined by Julayanont et al (2014), and (3) reported upon its diagnostic or prognostic utility within neurodegenerative dementia populations. Articles were excluded if they were not: (1) available in full text and (2) written in English.

We managed our systematic screening process using Covidence.^18^ After duplicate articles were automatically removed by this software, 2 authors (OK and AK) independently reviewed titles and abstracts to determine eligibility based on the above-outlined criteria. Any discrepancies were collaboratively resolved. Next, full-text articles were independently reviewed by 2 authors (OK and AK), and a third author (JS) resolved any differences. If provided, the following information was extracted from the included articles: authors, title, sample size, population, race, research aim(s), mean and variance of MoCA-TS, mean and variance of MIS, and results related to validity, reliability, or diagnostic accuracy. Data extraction was completed by one author (OK).

### Synthesis of Evidence

As heterogeneous methodologies precluded quantitative analysis, evidence was synthesized in a narrative format, organized below by clinical applications. A summary of the aims, sample size, and findings of the included studies is outlined in Table 2 to accompany the narrative review and for quick reference. In addition, participant characteristics (age, education, race, sex, MoCA TS, and MIS) for each study classified by diagnostic group are summarized in Table 3.

**Table 2.** Studies Examining the Clinical Utility of MoCA-MIS in Neurodegenerative Dementia Populations.

Study	Research aim	Setting	Findings
Predicting progression of cognitive decline			
Julayanont (2014)^5^	To evaluate the value of MoCA TS and MIS for predicting progression to AD in individuals with MCI	Community-based memory clinic in Montreal, Canada	• The TS and MIS had AUCs of 0.71 and 0.66, respectively, for predicting progression from MCI to AD over a mean 18.2 months of follow-up
			• The authors recommended an algorithm (a combined education-adjusted TS_cutoff_ < 20 + MIS _cutoff_<7), but this algorithm had limited sensitivity of 33%
Association with other measures			
Ang (2023)^27^	To validate MoCA domain scores with conventional neuropsychological tests	NACC database	• The MIS displayed weak to moderate correlations with measures of memory including craft story 21 immediate recall (*r* = 0.33), craft story 21 delayed recall (*r* = 0.43) and Benson complex figure recall (*r* = 0.42; *p*s < 0.001)
Kim (2021)^43^ a	To evaluate the association between MoCA index scores and corresponding SNSB-II scores	Outpatient neurology clinic in Anyang, South Korea	• The MIS displayed moderate correlations with the memory measure of the SNSB-II within the aMCI (*r* = 0.55) and VaMCI (*r* = 0.53) groups (both *p*s < 0.001), but only weak correlations within the AD (*r* = 0.22) and VaD (*r* = 0.24) groups (both *p*s > 0.05)
Ritter (2017)^38^	To investigate the correlation between MoCA memory scores and hippocampal volume	Outpatient neurology clinic in Las Vegas, Nevada, United States	• The free-recall score and MIS displayed weak to moderate correlations with hippocampal volume in the right (*r* = 0.36-0.39) and left (*r* = 0.39-0.40) hemispheres
Cayir (2025)^24^	To determine the association between MoCA scores and CSF biomarkers	Memory clinic in New Haven, Connecticut, United States	• In FTD, the MIS was not significantly correlated with CSF t-tau (*r* = −0.26) or p-tau_181_ (*r* = −0.14)
			• In AD, the MIS was moderately inversely associated with t-tau (*r* = −0.37, *P* < .05) but not p-tau_181_ (*r* = −0.25)
Differentiating between types of neurodegenerative disease			
Ang (2023)^27^	To explore whether MoCA domains scores can discriminate between etiologies in early NCD	NACC database	• The MIS was only associated with AD, with a relative risk of 1.15 (*P* < 0.001)^b^
			• The MIS had a relative risk of 1.0 for LBD and VD, and 1.01 for FTD (*P* > .05)^b^
Wood (2020)^28^	To evaluate the utility of MoCA index scores in differentiating between AD and PPA	NACC database	• The MIS predicted AD over PPA group membership with an odds ratio of 0.53, 95% CI 0.33-0.73
Differentiating between stages of cognitive decline			
Huang (2018)^32^ ^a^	To compare the MoCA-Basic, MIS, and a non-memory index score for differentiating NC, MCI, mild AD, and moderate AD	Memory clinic in Shanghai, China	• The MIS differentiated MCI vs NC with an AUCs 0.82-0.86 and had an optimal cutoff of <8, outperforming a MoCA score comprised of non-memory items (AUCs = 0.71- 0.77)
			• The MIS was less effective at differentiating MCI vs AD (AUCs = 0.72-0.74) and mild AD vs moderate AD (AUCs = 0.60-0.62), where non-memory items showed superior discrimination (AUCs = 0.84-0.90 and 0.79-0.83, respectively)
Dodge (2020)^23^	To examine utility of MoCA index scores in distinguishing among CDR scores	NACC database	• The MIS differentiated CDR scores with AUCs of 0.78 (0 vs 0.5), 0.95 (0 vs 1), and 0.76 (0.5 vs 1), which were similar to the TS (AUCs 0.80-0.97) and a more comprehensive composite of memory (AUCs 0.80-0.96)
			• When stratified by race, the AUC for the MIS was lower for Black (0.70) than white (0.80) participants at CDR 0 vs 0.5
Goldstein (2018)^33^	To evaluate MoCA index scores in their ability to differentiate between NC, MCI, and AD	ADNI database	• The MIS differed across NC vs MCI (Cohen’s *d* = 0.78), MCI vs AD (*d* = 1.09), and NC vs AD (*d* = 2.11) groups
			• In contrast, the TS also differed across NC vs MCI (*d* = 0.83), MCI vs AD (*d* = 1.61), and NC vs AD (*d* = 2.38) groups, with larger effect size
Kaur (2018)^37^	To compare MoCA-MIS and Craft Story 21 in differentiating NC from aMCI	NACC database	• The MIS (AUC = 0.83) and free-recall score (AUC = 0.82) was superior to Craft Story 21 (AUC = 0.80) in differentiating aMCI vs NC (*P* < 0.05)
Studies investigating free-, category cued-, and multiple-choice cued-recall performance independently			
Hari (2024)^20^	To investigate the association between MoCA memory subdomains and hippocampal volume	Community-based memory clinic in Nottingham, England	• The category-cued recall score showed a strong association with the dentate gyrus volume of the hippocampus (*r*_s_ = 0.63, *P* = 0.02)
			• The free-recall score showed a moderate, but non-significant, association with dentate gyrus volume (*r*_s_ = 0.44, *P* = 0.10)
Li (2018)^21^ ^a^	To determine the utility of the MoCA and its memory subdomain scores for identifying MCI subtypes	Memory clinics in Beijing, China	• The free (AUC = 0.78) and category-cued recall (AUC = 0.79) scores differentiated aMCI from NC similarly to the TS (AUC = 0.79). The memory subdomain scores displayed moderate positive association with RAVLT delayed recall (*r* = 0.37-0.50) and strong positive association with ROCF delayed recall scores (*r* = 0.51-0.61)
De Wit (2022)^19^	To evaluate whether the MoCA cued-recall items were additive in identifying episodic memory impairment	ADNI database	• Free-recall score significantly predicted episodic memory impairment (RAVLT score ≤2 SDs below the mean) with an odds ratio of 0.30, 95% CI 0.24-0.37
			• While controlling for free-recall, the addition of a cued-recall score improved model fit with an odds ratio of 0.67, 95% CI 0.58-0.77
Van Liew (2016)^16^	To explore whether the MoCA could differentiate memory subdomain deficits in NC, AD, and HD	Outpatient neurology clinics in San Diego, California, United States	• With free-recall, AD and HD groups were less likely to obtain points than NC (*p*s < .001) but did not differ from one another (*P* > .05)
			• With category cues, NC and HD groups displayed similar likelihood of obtaining points (*P* = 0.38) and both benefited more from cues than those with AD (*P* < 0.001)
			• With multiple-choice cues, NC were more likely to obtain points than HD and AD groups (*P* < .001), but HD benefited more from cues than those with AD (*P* = 0.002)

*Note*. MoCA = Montreal Cognitive Assessment; TS = MoCA Total Score, MIS = MoCA Memory Index Score; NACC = National Alzheimer’s Coordinating Center; SNSB-II = Seoul Neuropsychological Screening Battery–II; ADNI = Alzheimer’s Disease Neuroimaging Initiative; NCD = Neurocognitive Disorders; CSF = Cerebrospinal Fluid; T-tau = Total-tau; P-tau_181_ = phosphorylated tau at threonine 181; DR = Delayed Recall; PPA = Primary Progressive Aphasia; NC = Normal Control; CI = Confidence Interval; CDR = Clinical Dementia Rating; LBD = Lewy Body Dementia; VaD = Vascular Dementia; AD = Alzheimer’s Dementia; MCI = Mild Cognitive Impairment; aMCI = Amnestic MCI; VaMCI = Vascular MCI; HD = Huntington’s Disease; FTD = Frontotemporal Dementia; RAVLT = Rey Auditory Verbal Learning Test; ROCF = Rey-Osterrieth Complex Figure.

^a^A non-English version of the MoCA was utilized.

^b^Other etiologies of neurocognitive dementias (n = 832) were not included in the analysis and were rather used as a reference group.

**Table 3.** Participant Demographics and MoCA Performance Across Studies by Diagnostic/Etiologic Group.

Study (year)	Group	N	Age (M ± SD)	Education (M ± SD)	% white	% male	TS (M ± SD)	MIS (M ± SD)
Julayanont (2014)^5^	Non-converters	51	73.5 ± 1.1	11.1 ± 0.6	NR	46.6	22.1 ± 0.5	8.5 ± 0.4
	Converters	114	74.1 ± 0.7	10.3 ± 0.4	NR	53.4	19.3 ± 0.4	6.7 ± 0.3
Ang (2023)^27^	AD^a^	4418	NR	NR	NR	NR	NR	6.1 ± 3.8
	LBD^a^	428	NR	NR	NR	NR	NR	8.1 ± 3.9
	FTD^a^	602	NR	NR	NR	NR	NR	7.6 ± 4.7
	VaD^a^	304	NR	NR	NR	NR	NR	8.6 ± 4.0
	Overall	14,571	71.8 ± 8.9	16.0 ± 2.9	81.1	18.9	23.1 ± 5.4	NR
Kim (2021)^43^ ^b^	aMCI	104	73.7 ± 8.7	8.6 ± 4.5	NR	34.6	18.7 ± 5.0	4.6 ± 3.3
	VaMCI	74	73.7 ± 7.8	8.1 ± 4.7	NR	52.7	19.5 ± 4.6	5.6 ± 3.8
	AD	73	79.0 ± 11.0	7.9 ± 4.6	NR	31.5	14.1 ± 4.8	2.4 ± 1.9
	VaD	41	77.6 ± 6.0	8.6 ± 5.3	NR	46.3	13.1 ± 4.8	2.8 ± 3.0
Cayir (2025)^24^	FTD	28	64.1 ± 8.9	14.3 ± 3.4	NR	75.0	19.0 ± 6.5	7.7 ± 4.0
	AD	33	66.8 ± 8.8	14.7 ± 3.0	NR	39.4	17.0 ± 6.6	5.9 ± 3.8
Wood (2020)^28^	NC	83	75.9 ± 10.0	16.9 ± 2.3	NR	34.7	26.1 ± 1.9	12.0 ± 2.8
	AD	33	76.1 ± 9.9	16.0 ± 2.9	NR	49.1	18.9 ± 3.3	4.5 ± 2.9
	PPA	37	65.0 ± 6.7	16.3 ± 2.4	NR	54.0	21.0 ± 3.6	9.7 ± 4.4
Huang (2018)^32^ ^b^	NC	520	68.8 ± 8.2	10.7 ± 4.1	NR	34.2	24.1 ± 3.4	10.2 ± 6.6
	MCI	663	68.2 ± 8.5	10.8 ± 4.0	NR	46.0	19.4 ± 3.7	4.8 ± 3.9
	Mild AD	345	69.3 ± 8.8	10.2 ± 4.1	NR	47.0	14.4 ± 3.6	2.0 ± 2.9
	Moderate AD	441	69.2 ± 9.1	9.2 ± 4.3	NR	44.0	9.5 ± 3.7	1.0 ± 2.0
Goldstein (2018)^33^	NC	295	72.9 ± 6.0	16.6 ± 2.5	>90	54.9	25.6 ± 2.5	10.4 ± 3.5
	MCI	471	71.6 ± 7.5	16.1 ± 2.6	>90	45.4	23.2 ± 3.2	7.6 ± 3.9
	AD	150	74.7 ± 8.2	15.8 ± 2.7	>90	41.3	16.9 ± 4.5	4.1 ± 2.4
Dodge (2020)^23^	CDR 0	2274	71.2 ± NR	16.3 ± NR	76.4	34.7	25.9 ± NR	NR
	CDR 0.5	1722	72.6 ± NR	16.0 ± NR	82.2	49.1	22.1 ± NR	NR
	CDR 1	322	74.8 ± NR	15.5 ± NR	88.8	53.7	17.1 ± NR	NR
Kaur (2018)^37^	NC	2205	72.7 ± 10.0	16.3 ± 2.8	84.5	34.4	26.2 ± 2.9	12.2 ± 2.8
	aMCI	512	76.1 ± 9.2	16.0 ± 3.1	85.5	54.3	22.3 ± 3.5	7.8 ± 3.8
Ritter (2017)^38^	CC	138	70.7 ± 8.2	15.2 ± 2.7	89.9	50.7	21.9 ± 5.0	8.9 ± 4.1
Hari (2024)^20^	AD	24	60.0 ± NR	NR	NR	33.0	12.3 ± NR	NR
Li (2018)^21^ ^b^	amMCI	56	75.2 ± 7.1	14.0 ± 3.2	NR	44.6	20.9 ± 3.3	NR
	asMCI	32	71.0 ± 8.2	14.0 ± 2.7	NR	68.7	24.8 ± 2.2	NR
	naMCI	33	73.7 ± 7.7	13.8 ± 3.2	NR	48.5	23.8 ± 3.4	NR
	NC	53	70.2 ± 9.1	14.1 ± 2.4	NR	33.0	25.8 ± 2.3	NR
De Wit (2022)^19^	NC	719	71.8 ± 6.3	16.7 ± 2.4	86.7	40.8	26.1 ± 2.7	NR
	MCI	601	72.1 ± 7.5	16.1 ± 2.6	91.5	56.5	21.2 ± 4.5	NR
Van Liew (2022)^16^	NC	183	62.1 ± 16.5	15.3 ± 2.4	86.7	40.8	27.9 ± 1.9	NR
	HD	80	52.5 ± 14.2	14.3 ± 3.1	91.5	56.5	18.9 ± 5.9	NR
	AD	64	77.2 ± 8.1	15.8 ± 3.0	NR	NR	18.1 ± 5.6	NR

*Note*. NR = Not Reported; AD = Alzheimer’s Disease; MCI = Mild Cognitive Impairment; AMCI = Amnestic MCI; VAMCI = Vascular MCI; VAD = Vascular Dementia; CDR = Clinical Dementia Rating Global Score; FTD = Frontotemporal Dementia; HD = Huntington’s Disease; LBD = Lewy-Body Dementia; NC = Normal Control; PPA = Primary Progressive Aphasia; TS = MoCA Total Score; MIS = MoCA Memory Index Score; CC = Cognitive Complaint; amMCI = Amnestic Multi-Domain MCI; asMCI = Amnestic Single-Domain MCI; naMCI = Non-Amnestic MCI.

^a^The study grouped those with MCI and dementia.

^b^A non-English version of the MoCA was utilized.

## Results

### Selection of Sources of Evidence

**Figure 1.**
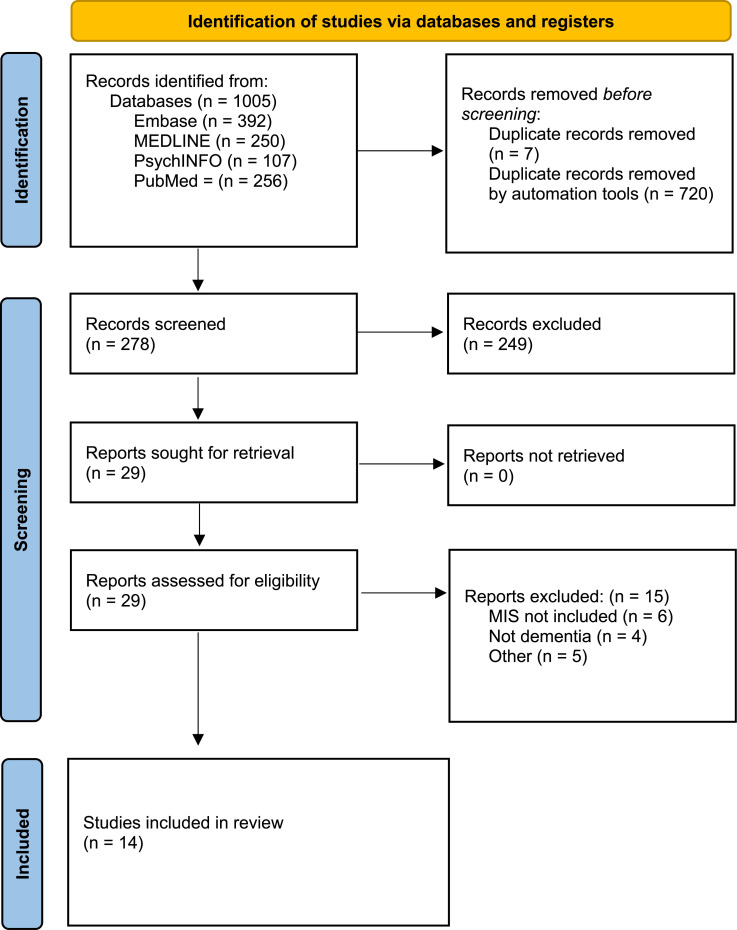
PRISMA flow diagram of database search.

Figure 1 illustrates the screening process and final studies identified from each source. Of the 278 studies initially identified, only 10 met all selection criteria. During the study selection process, 4 additional articles were identified that met most inclusion criteria, but did not evaluate the MIS as computed according to Julayanont et al (2014) guidelines.^16,19-21^ Rather, these 4 articles examined MoCA free- and cued-recall items independently rather than aggregated as the MIS. These articles were relevant to this review and are therefore included in the narrative synthesis below.

### Characteristics of Sources of Evidence

The majority of studies utilized English language versions of the MoCA (*k* = 11/14), while 2 used the Chinese and one used the Korean version. A large portion of studies reporting on the psychometric properties of the MIS relied on the National Alzheimer Coordinating Center (NACC) database (*k* = 4/14 of included studies utilized NACC samples). Study samples involved an overrepresented percentage of White (*k* = 7/7 studies that reported on race had >80% white participants) and highly educated subjects (*k* = 8/13 studies that reported education had mean >15 years). Furthermore, most studies investigating identifiability of diagnostic groups (*k* = 5/6, all but Huang et al [2018]) did not involve blinding or masking to MoCA scores which could bias the diagnostic process and result in artificially inflated agreement between MoCA scores and diagnostic group.^22^ Julayanont et al. (2014) attempted to account for this circularity and claimed that diagnosis was based primarily on functional decline rather than MoCA scores.^5^ However, ultimately, evaluators were not blinded to MoCA scores introducing the possibility of inflated diagnostic accuracy. Dodge et al. (2020) opted to use global Clinical Dementia Rating (CDR) scores to circumvent this circularity, as they claimed that CDR is not informed by neuropsychological test data.^23^ In a private communication with NACC, it was confirmed that CDR scores are indeed determined independently of neuropsychological data. Another methodological limitation was the use of suspected (ie, non-biologically derived) MCI and dementia etiology,^24^ which limits examination of the MIS predominantly to associations with dementia syndromes broadly rather than disease pathology. Finally, the version of the MoCA that was used was not reported. This is important to note as although the category structure is consistent (ie, all contain one word falling into body part, type of fabric, public place, type of food, and color categories) the stimulus words differ across versions, and research suggests that item factors within word list learning and memory tasks can impact performance.^25^ In addition, this limits our ability to establish test-retest reliability and alternate form reliability in clinical samples. In summary, 4 major limitations underlie existing psychometric data for the MIS, namely, (1) a reliance on non-diverse samples, (2) a lack of blinding to MoCA scores during the diagnostic process when evaluating MoCA diagnostic accuracy, (3) a lack of biologically derived etiology for cognitively impaired samples, and (4) a lack of reporting on the specific MoCA version utilized.

### Predicting Progression of Cognitive Decline

Although the MIS was initially added to enhance the predictive utility of the MoCA, surprisingly only one study has examined the scores longitudinally for this purpose.^4,5^ Namely, Julayanont et al (2014) suggested the use of an algorithm that combines an education-adjusted MoCA-TS cutoff of <20 and a MIS cutoff of <7, as the conversion rate was 90.5% for participants (n = 165) meeting both cutoffs during the 18-month follow-up. However, the stand-alone classification accuracy as measured by receiver operating characteristic (ROC) area under the curve (AUC) of the MIS was 0.66, while the MoCA-TS AUC was 0.71 and the orientation index score AUC was 0.70. Furthermore, results indicated that only 38 of the 114 converters were detected under the recommended combined cutoff algorithm conditions, suggesting the algorithm had low sensitivity in identifying converters. Using the reported results from the study, we calculated sensitivity and specificity and found that the suggested algorithm had an unacceptably low sensitivity of 0.33 but good specificity of 0.92 (Table 4).

**Table 4.** Classification of MCI to Alzheimer’s Dementia Converters Adapted From Julayanont et al (2014).^5^

		Actual conversion status		Total
		Converted	Stable	
Predicted conversion status	Converted	38	4	42
	Stable	76	47	123
	Total	114	51	165

*Note*. Data was extracted from Julayanont et al. (2014).^5^ In this article, a combined cutoff algorithm was recommended, using an education-adjusted TS_cutoff_ < 20 + MIS _cutoff_<7, to identify MCI to AD converters over short-term periods. In reporting their results, they emphasized that 90.5% (38/42; see row 1, predicted conversion) of individuals meeting both cutoffs were converters. However, the low sensitivity of 0.33 (38/114) was not highlighted in the manuscript. Thus, it should be noted that 76 of the 114 converters were not captured by this algorithm.

The authors proposed that sensitivity and specificity of the MIS for identifying MCI to AD converters could not be provided, as individuals within the normal range on MoCA scores were not followed up or included in the analysis. However, it is unclear why the sensitivity and specificity of the MIS and the combined algorithm was not provided within the specific context of an MCI memory clinic sample, a circumstance relevant to many clinicians and researchers assessing the likelihood that individuals with MCI will progress to develop AD. The low sensitivity is concerning given the use of the MoCA as a screening tool, as sensitivity is typically prioritized during screening to alert the need for comprehensive assessment.^26^ Further, the study had several noteworthy limitations, including potential bias, given that the primary developer of the MoCA was an author. Additionally, a relatively short (*M* = 18.2 months) and non-pre-established follow-up period was used. This means they were unable to assess the predictive ability of the MIS over longer periods. Relatedly, they likely missed individuals who would have converted over longer-term follow-up. Finally, as stated above, clinicians were not blinded to MoCA scores in assessing diagnosis, meaning that MoCA scores could have contributed to changes in diagnosis.

### Differentiating Between Neurodegenerative Diseases and Dementia Syndromes

Two studies using NACC samples have reported upon the differential utility of the MIS in assessing neurodegenerative diagnoses.^27,28^ However, it is important to emphasize that a limitation of this research is its lack of in vivo or postmortem biomarker consideration, which are becoming increasingly central to making neurodegenerative etiologic diagnoses.^29,30^ Therefore, this research fails to make a direct comparison between neurodegenerative-specific pathologies and the MIS. Rather, it relies on clinician diagnosis of clinical syndromes, again resulting in circularity.

Ang and colleagues (2023) explored the ability of MoCA domain-specific index scores, including the MIS, to discriminate between various etiologies in the early stages of neurocognitive disorders including individuals with MCI or dementia (n = 6584).^27^ Using multinomial logistic regression, findings supported the discriminative validity of the MoCA domain-specific index scores, as they were reflective of hallmark patterns observed in the most common forms of neurodegenerative dementias. Regarding the MIS, a relative risk ratio score of 1.15 for AD, 1.00 for Lewy body dementia, 1.01 for frontotemporal dementia, and 1.00 for vascular disease were observed, with AD being the only statistically significant result (*P* < 0.001). As expected, a profile of low memory and orientation domain-specific performance was most highly related to Alzheimer’s dementia.

Another study examined whether MoCA-TS, domain-specific scores, and the MIS could effectively differentiate between individuals classified as having normal cognition, AD, or a language-based dementia, Primary Progressive Aphasia (PPA).^28^ In the NACC sample, 153 individuals (n = 33 with mild AD, n = 36 with PPA, and n = 83 normal controls) completed the MoCA. Using a logistic regression model, they found that although MoCA-TS did not differ between AD and PPA patients, domain-specific index scores were different between the groups and helped differentiate by diagnosis. Specifically, findings suggested that higher MIS and orientation index scores predicted a lower likelihood of having an AD vs PPA diagnosis, with an MIS odds ratio of 0.53. These findings provide some support for the utility of domain-specific measures within the MoCA in differentiating diagnoses. Furthermore, they demonstrate that the MIS is sensitive to memory impairment, with individuals with diagnoses that are typified by memory impairment performing worse than those without central memory impairment.

### Differentiating Between Stages of Cognitive Decline

Four studies have reported on the MIS’s ability to differentiate between stages of cognitive decline. The first study by Huang et al. (2018) compared the MoCA-Basic (a modified MoCA incorporating items that are thought to be less influenced by education effects^31^), MIS, and an index of non-memory items in differentiating Chinese participants into normal cognition (n = 520), MCI (n = 663), mild AD (n = 345), and moderate AD (n = 520).^32^ Across low (≤6 years), mid (7-12 years), and high (>12 years) levels of education, the MIS differentiated MCI vs normal cognition with AUCs being 0.82-0.86 and an optimal cutoff of <8, outperforming a MoCA score comprised of non-memory items (AUCs 0.71-0.77, *P* < .001). However, the MIS was less effective at differentiating MCI vs AD (AUCs 0.72-0.74) and mild AD vs moderate AD (AUCs 0.60-0.62), where non-memory item showed superior discrimination (AUCs 0.84-0.90 and AUCs 0.79-0.83, respectively). The authors concluded that the MIS may be useful for detection of early stages of cognitive impairment, as delayed recall is often the first cognitive domain displaying measurable decline, whereas it appears ineffective at differentiating later stages of cognitive decline due to floor effects (both AD groups performed at floor levels with mean scores of 0.96 ± 2.04 and 1.99 ± 2.86).

A study by Goldstein et al. (2018) included participants with normal cognition (n = 295), MCI (n = 471), and AD (n = 150) from the Alzheimer’s Disease Neuroimaging Initiative (ADNI) database.^33^ Analysis of covariance was used to compare MoCA-TS, MIS, and domain-specific index scores in their ability to differentiate between cognitive stages. Effect sizes were interpreted as the differential ability between groups. The MoCA-TS demonstrated the largest effect sizes (*d* = 0.83 normal cognition vs MCI, *d* = 2.38 normal cognition vs AD, and *d* = 1.61 MCI vs AD), followed closely by the MIS (*d* = 0.78 normal cognition vs MCI, *d* = 2.11 normal cognition vs AD, and *d* = 1.09 MCI vs AD). A combined index score including the MIS, executive index score, and orientation index score was also evaluated, as these indices had the highest effect sizes and non-overlapping MoCA items. The MoCA-TS was outperformed by this combined index (*d* = 0.91 normal cognition vs MCI, *d* = 2.65 normal cognition vs AD, and *d* = 1.61 MCI vs AD). They reported that at an optimal cutoff of ≤23 points using the combined index, MCI and AD patients were classified with high sensitivity (0.92), specificity (0.90), and good overall classification accuracy as measured by AUC (0.87). In differentiating normal cognition from MCI, the combined index was not as effective, with the optimal cutoff of ≤27 resulting in a sensitivity of 0.73 and specificity of 0.62. They did not report on the sensitivity or specificity of the stand-alone MIS or TS.

The third study by Dodge et al. (2020) evaluated how MoCA scores differentiated between CDR scores in the NACC cohort, specifically among White and Black participants.^23^ They also reported on the classification accuracy of various other neuropsychological assessment instruments and a Memory Composite Score (including Immediate Craft Story Recall with paraphrase scoring, Delayed Craft Story Recall with paraphrase scoring, and total score for delayed recall of Benson figure), allowing for comparison between the MIS and other variables. They utilized global CDR scores of 0, 0.5, and 1, corresponding to normal, very mild dementia, and mild dementia, respectively.^34^

Results from Dodge et al (2020) showed that the MIS had AUC values of 0.78 in differentiating CDR 0 vs 0.5, 0.95 for CDR 0 vs 1, and 0.76 for CDR 0.5 vs 1. In comparison with the other MoCA domain-specific-index scores in the total sample, the MIS displayed the best classification accuracy at each stage, except for the MoCA orientation index more accurately classifying CDR 0.5 vs 1 (AUC = 0.79). In contrast, the MoCA-TS demonstrated superior classification accuracy when compared to the MIS (CDR 0 vs 0.5 AUC = 0.79, CDR 0 vs 1 AUC = 0.97, and CDR 0.5 vs 1 AUC = 0.81), suggesting that the MoCA-TS was a more useful tool overall. Similarly, the Memory Composite Score had better classification accuracy than the MIS across the groups (CDR 0 vs 0.5 AUC = 0.81, CDR 0 vs 1 AUC = 0.96, and CDR 0.5 vs 1 AUC = 0.80). However, when comparing the Memory Composite Score to the MIS, only the CDR 0 vs 0.5 had a significant difference, suggesting that the MIS performed similarly to the more comprehensive memory assessment in classifying CDR 0 vs 1 and CDR 0.5 vs 1. Notably, the MoCA-TS, MIS, and Memory Composite Score all displayed higher classification accuracy than a Global Composite Score, which included more comprehensive neuropsychological instruments assessing memory, language, attention, executive function, and visuospatial functioning (CDR 0 vs 0.5 AUC = 0.75, CDR 0 vs 1 AUC = 0.91, and CDR 0.5 vs 1 AUC = 0.72).

Importantly, findings from Dodge et al (2020) also suggested that racial differences may exist when differentiating individuals by CDR scores with the MIS. Within Black participants specifically, the findings were largely consistent, but some remarkable differences emerged. Namely, there was a significant difference between the CDR 0 vs 0.5 AUC values, with the MIS having lower accuracy in Black participants (AUC = 0.70) than in White participants (AUC = 0.80). This difference was not significant at other CDR score levels. Furthermore, the MoCA executive index showed higher accuracy in differentiating CDR 0 vs 1 (AUC = 0.95) and CDR 0.5 vs 1 (AUC = 0.85). Similarly, the MoCA orientation index more accurately differentiated CDR 0 vs 1 (AUC = 0.94) and CDR 0.5 vs 1 (AUC = 0.83). These differences indicate that the MIS may be less accurate in classifying early stages of cognitive decline in Black older adults. These discrepancies may be largely attributable to differences in educational attainment and quality. Therefore, normative educational adjustments to the MIS may help attenuate this inconsistency.

The fourth paper by Kaur and colleagues (2018) compared the MIS to Craft Story 21 delayed story recall, a measure which involves learning and remembering a short story after a 20-minute delay,^35,36^ for differentiating normal controls (n = 2205) from individuals with aMCI (n *=* 512) within a NACC sample.^37^ Uniquely, they also investigated 2 alternative MIS scoring methods, including free-recall alone (3 points per correct response) and free and category cued-recall (3 points per free-recall and 2 points per category cued correct response). While they did not find a difference between the 3 MIS scoring alternatives (AUC range from 0.82-0.83), they did find that the MIS (AUC = 0.83) was superior in identifying individuals with aMCI when compared to the Craft Story recall (AUC = 0.80; *P* = 0.004). At 80% sensitivity, the specificity for the MIS was 69.1% while Craft Story had a specificity of 62.8%. These findings suggest that the MIS may serve as a quick and effective tool for detecting aMCI, but they did not compare the MIS to the MoCA-TS, and they did not find a significant difference between the MIS and the conventional five-point free-recall score.

### Association with Other Neuropsychological Memory Measures

Regarding correlation with other neuropsychological memory variables, Ang et al. (2023; described above), reported on the construct validity of the MIS as it relates to other neurocognitive measures in the NACC dataset within a larger sample comprised of 14,571 individuals with normal cognition (n = 7987), MCI (n = 3534), and dementia (n = 3050).^27^ Using z scores of common neuropsychological assessment tools, the correlation between the MIS and visuospatial (Benson Complex Figure Copy), immediate memory (Craft Score 21 Immediate Recall), delayed memory (Craft Story 21 Delayed Recall and Benson Complex Figure Recall), language (Multilingual Naming Test and Verbal Fluency – L-Words), attention (Number Span Test Forward and Number Span Backward), processing speed (Trail Making Test Part A) and executive function (Trail Making Test Part B) were computed. Findings supported the notion that the MIS is more highly associated with memory than non-memory measures, but the correlations with memory measures were only weak to moderate. Specifically, correlations of *r* = 0.33 with Craft Story 21 Immediate Recall, *r* = 0.43 with Craft Story 21 Delayed Recall, and *r* = 0.42 with Benson Complex Figure Recall were observed (*P* < 0.001 for each memory measure). In comparison, the MIS demonstrated statistically significant but weak to negligible associations with non-memory measures. From highest to lowest, MIS correlated with the Multilingual Naming Test (*r* = 0.13), Trail Making Test Part B (*r* = 0.12), Trail Making Test Part A (*r* = 0.09), Verbal Fluency – L Words (*r* = 0.06), Number Span Test Forward (*r* = −0.05), Number Span Test Backwards (*r* = 0.04), and Benson Complex Figure Copy (*r* = −0.03).

Similarly, Kim et al (2021) evaluated the correlation between the MIS and the Seoul Neuropsychological Screening Battery, 2nd Edition (SNSB-II) in a Korean speaking population of participants with aMCI (n = 104), VaMCI (n = 74), AD (n = 73), and VaD (n = 41). They found that the MoCA MIS displayed moderate correlations with the memory measure of the SNSB-II within the aMCI (*r* = 0.55) and VaMCI (*r* = 0.53; both *P*-values <.001) groups, but only weak correlations within the AD (*r* = 0.22) and VaD (*r* = 0.24; both *P*-values >.05) groups. They suggested that this discrepancy and weak association may be a result of floor effects on the MIS, which was notable in patients with AD (2.42 ± 1.86) and VaD (2.76 ± 2.97).

### Association with Anatomical Correlates of Memory

Some evidence exists supporting an association between the MIS and neuroanatomical structures implicated in memory. One study investigated the association between the MIS scores and hippocampal volume in a mixed clinical sample of 138 individuals presenting to a clinic with memory complaints.^38^ Ritter and colleagues also investigated the performance on delayed-recall items alone, enabling evaluation of whether cued-recall was additive in this association. Findings supported the hypothesis that lower scores on the MIS and MoCA delayed-recall items were weakly to moderately associated with lower hippocampal volume in both the left and right hemispheres; however, there was not a statistically significant difference between the 2 MoCA memory scores. The association of MIS scores was similar for left hippocampus (*r* = 0.40; LH) and right hippocampus (*r* = 0.39; RH) volumes, and resembled correlations between MoCA delayed-recall and LH (*r* = 0.39) and RH (*r* = 0.36) volumes. When accounting for demographic variables, regression analysis R-squared values of the MoCA delayed-recall were 0.12 in the LH and 0.11 in the RH. The MIS demonstrated an R-squared value of 0.13 in both hemispheres. These findings suggest that low MoCA memory scores may reflect hippocampal atrophy; however, they do not provide evidence for an advantage of the MIS over free-recall items alone. The authors suggested that this may be a result of the heavy weighting of free-recall performance within the MIS, resulting in minor difference between the 2 indices. Therefore, they suggest that a calculation of a dedicated cued-recall performance score, particularly when free-recall is at floor levels, might be more useful. However, there has yet to be an investigation validating the utility of such a score.

### Association with Neuropathological Markers of Neurodegenerative Disease

Cayir et al (2025) analyzed the association of the MoCA TS and MIS with cerebrospinal fluid-derived tau markers in 61 patients with AD (n = 33) and FTD (n = 28). In the FTD group, total-tau (t-tau) was significantly inversely moderately associated with the MoCA TS (*r* = −0.47, *P* = .01) but not the MIS (*r* = −0.26, *P* > .05). In contrast, in AD patients, t-tau was inversely moderately correlated with the TS (*r* = −0.54, *P* < .01) and weakly correlated with the MIS (*r* = −0.37, *P* < .05). The MIS was not significantly correlated with phosphorylated tau at threonine 181 (p‐tau_181_) in either group, whereas the TS was inversely moderately correlated with p‐tau_181_ in the AD (*r* = −0.55, *P* < .01) but not the FTD (*r* = −0.22, *P* > .05) groups. Overall, the findings suggest that the TS is more strongly associated with t-tau and p‐tau_181_ markers with well-established associations to cognitive decline. However, the authors suggested that the lack of association between the MIS and t-tau levels in the FTD group could be due to preserved memory function in early stages of FTD, which could have implications for differentiating dementia syndromes (ie, lower MIS may be more reflective of AD over FTD). However, the ability of MoCA scores to differentiate the diagnostic groups was not specifically investigated and there was significant overlap in the distribution of MIS scores across the groups.

### Studies Investigating Free-, Category Cued-, and Multiple Choice Cued-Recall Performance Independently 

During the search process, 4 studies were identified that investigated the memory components underlying the MIS, but these studies did not report on the MIS as scored using the Julayanont et al. (2014) MIS paradigm.^5^ They are included here as they report information relevant to the utility of the MIS in neurodegenerative populations and are therefore relevant to the aims of this review. The first of the studies reported upon the association of MoCA memory scores and hippocampal volume. Hari and colleagues (2024) investigated the association between MoCA memory subdomains and hippocampal volume as measured by T7 MRI in various regions of interest within the hippocampus in individuals who were diagnosed with probable Alzheimer’s disease (n = 24) recruited from local memory clinics.^20^ They examined associations between MoCA memory scores and the cornu ammonis (CA) areas (CA1, CA2, and CA3), the dentate gyrus, entorhinal cortex, the hippocampal tail, and the subiculum using Spearman’s correlations. After correcting for multiple comparisons, their findings demonstrated a significant association between the dentate gyrus and category-cued memory performance (*r*_s_ = 0.63, *P* = 0.02), while all other associations did not hold up to correction for multiple comparisons. A critical limitation, however, is that there was no acknowledgement that the MoCA memory assessment paradigm uses a step-down procedure, meaning that cued memory performance may be inversely associated with free-recall memory performance. Therefore, examining correlations between hippocampal volume and scores on MoCA category-cued and multiple-choice-cued-recall as if performance is independent is not possible. Free-recall performance, given that it proceeds cues, is the most directly interpretable. Although non-significant, results suggest that correlations between hippocampal regions and MoCA free-recall, from highest to lowest, proceed as follows: dentate gyrus (*r*_s_ = 0.44, *P* = 0.10), the hippocampal tail (*r*_s_ = 0.33, *P* = 0.27), CA1 (*r*_s_ = 0.33, *P* = 0.25), subiculum (*r*_s_ = 0.25, *P* = 0.45), CA3 (*r*_s_ = 0.24, *P* = 0.42), the entorhinal cortex (*r*_s_ = 0.20, *P* = 0.45), and CA2 (*r*_s_ = −0.11, *P* = 0.63). Together, these findings suggest that the volume of the dentate gyrus, theorized to be implicated in various memory processes such as discriminating memories, unifying sensory inputs, detecting novelty, maintaining specificity by filtering only strong signals, supporting the formation of engrams, and indexing time and context of learning,^39^ is particularly associated with MoCA memory performance, but additional research is needed to understand the association between this region and the MIS.

Li et al. (2018) investigated the Beijing version of the MoCA and its memory subdomain scores in differentiating single-domain aMCI (n = 56), multi-domain aMCI (n = 32), and non-amnestic MCI (n = 33) from those with normal cognition (n = 53), while also reporting on the association with Rey Auditory Verbal Learning Test (RAVLT) and the Rey-Osterrieth complex figure (ROCF) delayed recall scores.^21^ Importantly, the authors reported the category and multiple-choice cued-recall scores but did not clarify how these scores were derived (ie, simply stating 0-5 points without specifying how it was scored if the word was recalled accurately at the prior recall level). In identifying single- or multi-domain aMCI, the free-recall (AUC = 0.78) and category cued-recall (AUC = 0.79) were similar to the MoCA TS (AUC = 0.79) but were inferior to the RAVLT (AUC = 0.91) and ROCF (AUC = 0.87). Although, it is important to acknowledge that the RAVLT and/or ROCF scores were used to classify participants as having aMCI. The multiple-choice cued-recall had the lowest discrimination (AUC = 0.69) of aMCI from normal cognition. As may be expected, a notable strength of the MoCA memory subdomain scores over the TS was in identifying single-domain aMCI (AUC values for TS = 0.63 vs free-recall = 0.72 and category cued-recall = 0.69), suggesting they may be more effective in identifying focal memory deficits. AUC values were lower for differentiating non-aMCI from normal cognition for all the MoCA memory subdomain scores (AUCs = 0.56-0.69) and the TS (AUC = 0.69). Notably, the MoCA memory subdomain scores showed moderate positive association with the RAVLT (Pearson’s *r* = 0.37-0.50) and strong positive association with the ROCF (Pearson’s *r* = 0.51-0.61), with the category cued-recall having the strongest correlation.

De Wit and colleagues (2022) reported upon the added utility of cued-memory items of the MoCA in detecting memory impairment.^19^ Using data from the Alzheimer’s Disease Neuroimaging Initiative, they examined how the MoCA memory scores predicted impaired memory (classified by a RAVLT score 2 standard deviations below the mean) in a sample of 719 normal controls and 601 individuals with MCI. They used the free-recall score (ranging from 0-5 points) and a combined MoCA cued-recall score (also ranging from 0-5 points, with 1 point for *either* a correct free-recall, category cued-recall, or multiple-choice cued-recall, ie, the participant was able to recall the word at any stage). They found that, in a stepwise logistic regression, cued-recall and multiple-choice cued-recall performance improved the overall classification beyond free-recall and participant demographics alone. More specifically, they found that for each point increase in the MoCA free-recall, the odds of having impaired memory decreased by 70%, OR = 0.30, 95% CI = 0.24, 0.37. For the combined free/cued-recall score, the odds for being in the impaired group decreased by 33%, OR = 0.67, 95% CI = 0.58, 0.77, for each one-unit increase. These results suggest that adding cued-recall performance to the free-recall score has some additive value in predicting impaired RAVLT scores.

Another study evaluated the MoCA memory subdomains independently using maximum likelihood models.^16^ They sought to assess performance on free-recall, category cued-recall, and multiple-choice cued-recall items in a sample of 327 individuals with Huntington’s Disease (HD; n = 70), AD (n = 64), and normal controls (n = 183) from California dementia research center samples. They observed that patterns of memory performance differed between the HD, AD, and normal control groups. While controlling for age and education, they found that participants with AD and HD both performed worse on the free-recall items of the MoCA than the normal controls, but the AD and HD participants displayed no difference from one another. In contrast, with category cueing, the normal controls and HD participants both benefited from cues, while the AD participants did not. Finally, on the multiple-choice cued-recall items participants with HD and AD performed worse than the normal controls, while individuals with HD were more likely to correctly respond than those with AD. This study supports the notion that the multidimensional memory items of the MoCA have utility for differentiating dementia etiologic diagnoses, indirectly offering support for the MIS for this purpose. However, the authors explained that the detail provided by examining performance at each level independently may be lost with an average across all levels, as is the case with the MIS.

## Discussion

This comprehensive review suggests the MIS can add incremental validity to the MoCA as there is some empirical support for select use cases. However, the available evidence on the clinical utility of the MIS is sparse. Various gaps in the literature were identified, including limitations with methodological rigor (eg, lack of blinding to MoCA scores when evaluating diagnostic utility and lack of reporting on MoCA version utilized) and non-diverse research samples with heavy reliance on the NACC cohort. Therefore, future research using diverse community samples is needed to better evaluate the psychometric properties of the MIS and inform its utility in non-White individuals. While some studies provide normative data on the MIS for specific populations, such as Dutch, Brazilian, and Italian samples, additional research from other representative community standardization samples would enhance the inferences that could be derived from the MIS and lay the groundwork for wider application.^40-42^

Despite early claims that the MIS may be a useful predictor of progression from MCI to AD,^4^ only one study has investigated this particular use.^5^ The findings of this sole investigation were limited by a short follow-up period and a lack of reporting on sensitivity and specificity with the studies suggested algorithm displaying very low sensitivity for predicting MCI to AD progression as was highlighted in this review. Thus, evidence supporting this claim is currently unsubstantiated.

Regarding the broader utility of the MIS, published research provides initial support for its use in neurodegenerative dementia populations, as the MIS shows weak to moderate correlations with neuropsychological memory measures and patterns of hippocampal atrophy typically seen in this clinical group.^21,27,38^ Furthermore, the MIS may aid in differentiating dementia syndromes and stages of cognitive decline.^21,23,27,28,33^ However, it is noteworthy that the MoCA-TS outperformed the MIS as a stand-alone measure for classifying stages of dementia progression in the examined studies, while the MIS can theoretically help identify specific cognitive deficits in memory to aid in assessing different clinical syndromes with poor recall. A major limitation of the MIS is its narrow range, resulting in potential ceiling effects in healthy individuals and floor effects in populations with dementia, although scores differed substantially across studies (eg, 0.96 ± 2.04 in the “moderate AD sample” in Huang et al [2018] vs 5.88 ± 3.8 in the AD sample from Cayir et al [2025]).^32,43^ These findings suggest that the MIS may be a valuable supplement to TS but is clearly not a substitute for comprehensive memory assessment as part of a neuropsychological evaluation. Therefore, the MIS should be interpreted within the context of additional assessment data and clinical information when available.

This review is unable to sufficiently answer several important questions that arise regarding the MIS. Firstly, the added value of cued-recall items within the MoCA is unclear (ie, how does the MIS differ from delayed free-recall score, and does it warrant the extra time required). Only 2 of the included studies directly compared the MIS to the conventional five-word delayed-recall score.^37,38^ In addition, 4 studies were identified that investigated free-, category cued-, and multiple-choice cued-recall on the MoCA; however, the studies did not follow the MIS scoring recommendations and thus did not allow direct comparisons between independent subdomains scores and the MIS aggregate.^16,19,20^ Although limited, some evidence does suggest that MoCA cued-recall items improve identification of memory impairment above and beyond delayed free-recall performance.^19^ Moreover, on average, performance on the various memory subdomains of the MoCA does differ between clinical diagnoses, suggesting that differential assessment of specific memory deficits may be possible.^16^ These findings lend support to the notion that the MIS adds incremental validity to the MoCA beyond the conventional five-word recall score, but additional research is needed to understand the clinical value of this difference.

Another important question that cannot be conclusively answered currently is how future iterations of the MoCA might improve its memory components to increase its utility in neurodegenerative dementias. The MIS step-down cues provide aid to retrieval while simultaneously assessing memory subdomains, ultimately helping reduce floor effects which are common on the MoCA in amnestic populations.^32,43,44^ Nevertheless, this step-down procedure and scoring paradigm comes with limitations. Performance at a prior level subsequently affects performance at the following level. For example, if an individual fails to freely recall any of the 5 words, they will have more opportunities to obtain points during the cued-recall. This limits one’s ability to assess cued-recall independently of free-recall performance. In addition, the point value assigned to each level of the MIS appears to be selected based on difficulty (with 3 points for free-recall being the most difficult, and one point for multiple-choice cued-recall being easiest). However, it is not clear whether the proportional difference between these values reflects meaningful differences clinically. Moreover, as expressed by Ritter et al. (2017), free-recall performance is heavily weighted in the MIS.^38^ Ultimately, assessing memory performance collectively with the MIS, rather than examining performance at each stage individually, limits the nuance of information that can be extracted.^16^ Thus, modification of the scoring model of the MIS, the general structure of the MoCA memory measures, or future iterations of neurodegenerative dementia screening measures may be warranted. To this end, a clearly defined goal of the MIS is necessary. If the goal is indeed to predict progression to AD in MCI subjects as stated by Julayanont and Nasreddine (2017), then alterations may be warranted given the questionable results of the single investigation.^4,5^

While our review focuses on the clinical utility of the MIS, it is important to recognize that this score is not typically interpreted in isolation in clinical settings. Rather, the clinical determination of diagnosis or prognosis requires a comprehensive evaluation, factoring in a wide variety of confirmed diagnostic features and/or risk factors. Moreover, the MIS was developed as a brief screening tool and not through rigorous psychometric development methods (eg, item response theory, item internal consistency, or identifying ideal items through larger item pools). According to Julayanont and Nasreddine (2017), the MoCA was developed using “clinical intuition” to screen for MCI and later the MIS was added “to predict AD conversion among patients with MCI.”^4^ However, this prognostic MIS application has not been validated through the existing literature. In addition to altering the task procedures, the MoCA may further improve its diagnostic and prognostic utility through robust empirical, rather than intuition-based, psychometric item development.

Furthermore, additional evidence demonstrating the test-retest reliability, reliable change, and sensitivity to change in clinical populations would strengthen evidence for the utility of this brief tool. Only one study investigated the test-retest reliability of the MIS which did not include a clinical sample. In a Dutch sample, the intra-class correlations across MoCA versions 7.1, 7.2, and 7.3 were poor between 7.1 to 7.2 (0.32) and fair for 7.1 to 7.3 (0.48), indicating a reliable change score of ±3.4 and ±3.0, respectively, after a mean interval of 22.1 days.^45^ This suggests that there is substantial variability across versions, even within healthy participants over a short follow-up period. Therefore, a MIS change of greater than 3 or 4 points is needed to affirm statistical significance in this sample of healthy individuals which is a large portion of the overall range, and replication in cognitively impaired groups across MoCA versions and translations is needed.

This review, along with the broader literature on this topic, has several noteworthy limitations. First, as we have outlined, much of the investigation has utilized NACC data, meaning that several of these studies may have examined MoCA scores in overlapping subjects limiting the diversity represented in the MIS literature. Second, no formal risk-of bias or critical appraisal protocols were followed to assess the quality of included studies, and we are therefore unable to assure the quality of the research examined in this review. Third, it is possible that this review overlooked studies reporting on the clinical utility of the MoCA memory subdomains, as we did not aim to comprehensively capture all available literature on the MoCA free, category-cued, or multiple-choice cued-recall memory domains independently but rather as aggregated in the MIS. Fourth, much of the existing research on the MIS has limited interpretability, as studies report statistically significant differences between group or within regression models in large cohort studies rather than providing diagnostic accuracy statistics.^46^ Finally, this review is limited by the available evidence on this topic. Despite broad inclusion criteria and search strategy, only 14 studies were ultimately retained and included in this review, some of which included the MIS as only a peripheral variable. These factors limit these findings and highlight the need for additional research on the MIS.

Future studies should address the limitations of the literature highlighted in this review. Firstly, research within diverse racial, ethnic, and diagnostic populations is needed. In addition, future research on the diagnostic or prognostic utility of the MIS should report results using clinically interpretable statistical methods, such as sensitivity, specificity, and positive/negative predictive values. Furthermore, research investigating the diagnostic utility of the MIS should ideally blind clinicians to MoCA scores during diagnostic consideration, as this would remove potential circularity involved in comparing MoCA scores to diagnosis. Relatedly, the association between the MIS and other measures, such as functional decline, collateral reports, in vivo and post-mortem neurodegenerative biomarkers, and functional and anatomical neuroimaging must be explored to extend understanding of how the MIS relates to features of neurodegenerative disease. Critically, if the goal of the MIS is to predict conversion from MCI to AD as suggested by Julayanont and Nasreddine (2017), longitudinal research over longer-term follow-up is needed to substantiate claims that it is an effective tool for identifying MCI to AD converters.^4^ In doing so, it may be necessary to explore the scoring and task procedures of the MIS, tailoring them to the desired goal.

In conclusion, the MIS shows some promise as a supplemental tool within MoCA screening. However, research to date is limited, and well-substantiated conclusions cannot be made regarding its overall clinical utility within neurodegenerative dementias. The MIS provides novel information about cued-recall performance, with some findings suggesting value above and beyond the TS for detection of memory impairment and aiding in differentiation of dementia syndromes. Evidence supports the notion that the MIS correlates with expected neurocognitive variables and has potential to contribute to addressing various clinically relevant questions. Nevertheless, as a stand-alone measure, existing literature does not validate the MIS for differentiating between stages of cognitive decline, differentiating dementia etiologies, nor predicting MCI to AD progression at present. Therefore, the MIS should be interpreted within the context of the TS and more comprehensive neuropsychological assessment.

## Supplemental Material

**Supplemental Material -** A Scoping Review of Clinical Utility From the Montreal Cognitive Assessment Memory Index Score Supplemental Material for A Scoping Review of Clinical Utility From the Montreal Cognitive Assessment Memory Index Score by Oscar R. Kronenberger, Alyssa N. Kaser, Jeffrey Schaffert^1^, Vishal J. Thakkar, William Goette, Christian LoBue, and Laura H. Lacritz in Journal of Geriatric Psychiatry and Neurology.
