# Supplementary material for: A Scoping Review of Clinical Utility from the Montreal Cognitive Assessment Memory Index Score
Source: J Geriatr Psychiatry Neurol. 2025 Aug 18;39(3):261–76. doi: 10.1177/08919887251366698 (PMC13009243; doi:10.1177/08919887251366698)
Supplement: Supplemental Material - A Scoping Review of Clinical Utility From the Montreal Cognitive Assessment Memory Index Score [file sj-pdf-1-jgp-10.1177_08919887251366698.pdf]

Supplemental Table 1: Search String and Results from Databases

Database	String Search
Medline	(("cognitive decline" or "cognitive impairment" or "mild cognitive impairment" or "dementia" or "neurodegenerative" or "amnestic" or "AD" or "MCI" or "FTD" or "LBD" or "sensitivity" or "specificity" or "classification accuracy" or "ROC" or "receiver operating characteristic" or "neuropsych*" or "neuroimaging" or "brain imaging" or "neurological" or "predictive value" or "alzheimer*" or "vascular" or "frontotemporal" or "lewy bod*" or "psychometrics" or "validity" or "reliability").ti,ab,kf.) OR (dementia/ or alzheimer disease/ or dementia, vascular/ or frontotemporal lobar degeneration/ or lewy body disease/ or mixed dementias/ or cognition disorders/ or cognitive dysfunction/ or neurocognitive Disorders/ or reproducibility of results/ or "sensitivity and specificity"/ or predictive value of test/ or prognosis/ or psychometrics/ or neuropsychological test/ or neuroimaging/ or functional neuroimaging/)) AND (("Montreal Cognitive Assessment" or "MoCA").ti,ab,kf.) AND ("memory index score" or "MIS" or "domain score*" or "domain-specific score*" or "index score*").ti,ab,kf.))
Embase	(("cognitive decline" or "cognitive impairment" or "mild cognitive impairment" or "dementia" or "neurodegenerative" or "amnestic" or "AD" or "MCI" or "FTD" or "LBD" or "sensitivity" or "specificity" or "classification accuracy" or "ROC" or "receiver operating characteristic" or "neuropsych*" or "neuroimaging" or "brain imaging" or "neurological" or "predictive value" or "alzheimer*" or "vascular" or "frontotemporal" or "lewy bod*" or "psychometrics" or "validity" or "reliability").ti,ab,kf.) OR (dementia/ or alzheimer disease/ or dementia, vascular/ or frontotemporal lobar degeneration/ or lewy body disease/ or mixed dementias/ or cognition disorders/ or cognitive dysfunction/ or neurocognitive Disorders/ or reproducibility of results/ or "sensitivity and specificity"/ or predictive value of test/ or prognosis/ or psychometrics/ or neuropsychological test/ or neuroimaging/ or functional neuroimaging/)) AND (("Montreal Cognitive Assessment" or "MoCA").ti,ab,kf.) AND ("memory index score" or "MIS" or "domain score*" or "domain-specific score*" or "index score*").ti,ab,kf.))
PsychINFO	((("cognitive decline" or "cognitive impairment" or "mild cognitive impairment" or "dementia" or "neurodegenerative" or "amnestic" or "AD" or "MCI" or "FTD" or "LBD" or "sensitivity" or "specificity" or "classification accuracy" or "ROC" or "receiver operating characteristic" or "neuropsych*" or "neuroimaging" or "brain imaging" or "neurological" or "predictive value" or "alzheimer*" or "vascular" or "frontotemporal" or "lewy bod*" or "psychometrics" or "validity" or "reliability").ti,ab,id.) OR (dementia/ or neurocognitive disorders/ or alzheimer's disease/ or dementia with lewy bodies/ or frontotemporal lobar degeneration/ or vascular dementia/ or cognitive aging/ or cognitive impairment/ or mild cognitive impairment/ or neurodegenerative diseases/ or prognosis/ or psychometrics/ or neuroimaging/ or cognitive assessment/ or screening tests/ or test reliability/ or test validity/ or psychometrics/ or Neuropsychological Assessment/)) AND (("Montreal Cognitive Assessment" or "MoCA").ti,ab,id.)) AND (("memory index score" or

	"MIS" or "domain score*" or "domain-specific score*" or "index score*").ti,ab,id.))
PubMed	(("Mental Status and Dementia Tests"[MeSH Terms] OR "Dementia"[MeSH Terms] OR "Alzheimer Disease"[MeSH Terms] OR "Cognition Disorders"[MeSH Terms] OR "dementia, vascular"[MeSH Terms] OR "Frontotemporal Lobar Degeneration"[MeSH Terms] OR "Lewy Body Disease"[MeSH Terms] OR "Mixed Dementias"[MeSH Terms] OR "Cognitive Dysfunction"[MeSH Terms] OR "Neurocognitive Disorders"[MeSH Terms] OR "Reproducibility of Results"[MeSH Terms] OR "Diagnostic Techniques and Procedures"[MeSH Terms] OR "Psychometrics"[MeSH Terms] OR "Neuropsychological Tests"[MeSH Terms] OR "Neuroimaging"[MeSH Terms] OR "Functional Neuroimaging"[MeSH Terms] OR ("cognitive decline"[Title/Abstract] OR "cognitive impairment"[Title/Abstract] OR "mild cognitive impairment"[Title/Abstract] OR "Dementia"[Title/Abstract] OR "neurodegenerative"[Title/Abstract] OR "amnestic"[Title/Abstract] OR "AD"[Title/Abstract] OR "MCI"[Title/Abstract] OR "FTD"[Title/Abstract] OR "LBD"[Title/Abstract] OR "sensitivity"[Title/Abstract] OR "specificity"[Title/Abstract] OR "classification accuracy"[Title/Abstract] OR "ROC"[Title/Abstract] OR "receiver operating characteristic"[Title/Abstract] OR "neuropsych*"[Title/Abstract] OR "Neuroimaging"[Title/Abstract] OR "brain imaging"[Title/Abstract] OR "neurological"[Title/Abstract] OR "predictive value"[Title/Abstract] OR "alzheimer*"[Title/Abstract] OR "vascular"[Title/Abstract] OR "frontotemporal"[Title/Abstract] OR "lewy bod*"[Title/Abstract] OR "Psychometrics"[Title/Abstract] OR "validity"[Title/Abstract] OR "reliability"[Title/Abstract])) AND (("memory index score"[Title/Abstract] OR "MIS"[Title/Abstract] OR "domain score*"[Title/Abstract] OR "domain specific score*"[Title/Abstract] OR "index score*"[Title/Abstract]) AND ("Montreal Cognitive Assessment"[Title/Abstract] OR "MoCA"[Title/Abstract]))) AND (2014:2024[pdat])

Note. The search was conducted in July 2025.
